# Biomarkers in Cardiorenal Syndrome

**DOI:** 10.3390/jcm10153433

**Published:** 2021-07-31

**Authors:** Giovanni Goffredo, Roberta Barone, Vito Di Terlizzi, Michele Correale, Natale Daniele Brunetti, Massimo Iacoviello

**Affiliations:** 1 Department of Medical and Surgical Sciences, University of Foggia, Viale Luigi Pinto 1, 71122 Foggia, Italy; giovannigoffredo1993@gmail.com (G.G.); barone.r90@gmail.com (R.B.); vitodt89@gmail.com (V.D.T.); michele.correale@libero.it (M.C.); natale.brunetti@unifg.it (N.D.B.); 2 Cardiology Unit, University Policlinic Hospital Riuniti, Viale Luigi Pinto 1, 71122 Foggia, Italy

**Keywords:** heart failure, renal function, cardio-renal syndrome, acute kidney injury, biomarkers

## Abstract

Cardiorenal syndrome is a clinical manifestation of the bidirectional interaction between the heart and kidney diseases. Over the last years, in patients with cardiovascular diseases, several biomarkers have been studied in order to better assess renal function as well as to identify patients prone to experiencing chronic or acute worsening of renal function. The aim of this review is to focus on the possible clinical usefulness of the most recent biomarkers in the setting of cardiorenal syndrome.

## 1. Introduction

In patients affected by cardiovascular diseases (CVD), chronic kidney disease (CKD) is frequently present. The prevalence of an altered renal function is even greater when patients affected by both acute and chronic heart failure (HF) are considered [1,2,3].

This relationship is due to the bidirectional interaction between the heart and kidneys, i.e., two organs sharing both physiological and pathological conditions [2]. On one hand, the heart is considerably dependent on fluid homeostasis, regulated by the kidney, whereas, on the other hand, renal function is subordinated to blood perfusion, regulated by hemodynamic, neurohormonal, inflammatory and local mechanisms [4,5]. Moreover, heart and kidney diseases frequently coexist because of sharing risk factors (such as hypertension, diabetes and atherosclerosis) as well as common pathophysiological pathways [2]. In fact, aside from hemodynamic factors, neurohormonal overactivity, endothelial dysfunction, inflammation, oxidative stress are all conditions able to favor the progression of both CVD and CKD in acute and chronic settings [2].

The close relationship between cardiovascular and renal diseases and the possibility of a reciprocal influence in determining their progression has been defined as cardiorenal syndrome (CRS) [2]. CRS has been further distinguished in five different subtypes according to the organ which is mainly responsible of the worsening of the other one. Type 1 refers to the acute kidney injury (AKI) caused by acute cardiac disease; type 2 to chronic kidney disease (CKD) caused by chronic heart disease; type 3 to the heart dysfunction caused by the acute worsening of kidney function; type 4 to cardiac disease determined by CKD; finally, type 5 is characterized by a simultaneous injury of the heart and kidneys caused by systemic diseases [2].

**Figure 1:**
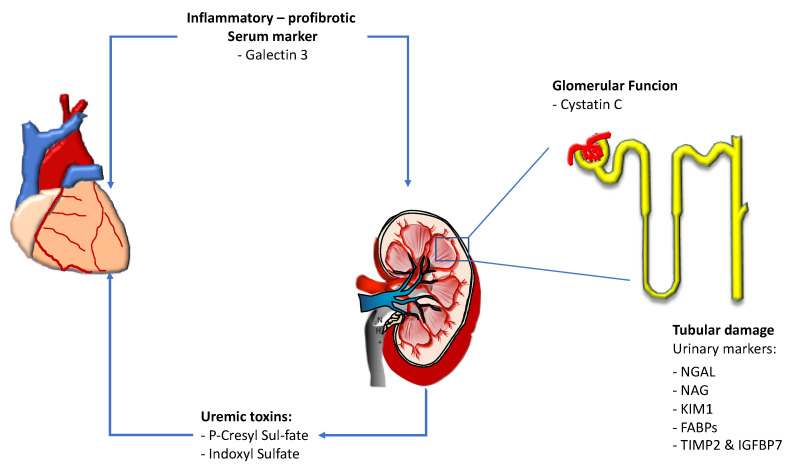
Main renal biomarkers indicative of cardiorenal and renocardiac syndrome progression. NGAL: N-acetyl beta glucosaminidase. NAG: N-acetyl beta glucosaminidase. KIM 1: Kidney injury molecule. FABPs: Fat-ty acid-binding proteins; TIMP2 & IGFBP7: Tissue inhibitor of metalloproteinase 2 and insulin-like growth factor–binding protein 7.

Over the last years, several biomarkers have been studied in order to better evaluate the severity of renal dysfunction as well as to accurately identify early on the risk of cardiorenal syndrome progression (Figure 1, Table 1) [4]. These biomarkers are related to the different pathophysiological aspects of cardiorenal syndrome, from markers of systemic inflammation to those reflecting glomerular and tubular function, until those related to end stage renal disease such as uremic toxins.

The aim of this review is to focus on the available biomarkers and on their possible clinical usefulness in the setting of cardiorenal syndrome.

## 2. Renal Biomarkers in Chronic Kidney and Cardiovascular Diseases

Renal function is generally assessed by formulas which estimate glomerular filtration rate (GFR) on the basis of serum creatinine levels [5]. Serum creatinine is the results of skeletal muscles creatine phosphate breakdown [6]. Its rate of production is relatively constant and its elimination by the kidney is mainly mediated by glomerular filtration and only partially by an active tubular secretion [6]. Over the last decades, empirical formulas have been demonstrated to easily and accurately estimate GFR on the basis of serum creatinine levels, age, gender, weight and race [7,8,9]. Cockroft–Gault, simplified Modification of Diet in renal disease (MDRD) and, more recently, Chronic Kidney Disease Epidemiology Collaboration (CKD EPI) [5,7,8,9] are the most commonly used formulas. In comparison with the other formulas, particularly in patients with preserved renal function, CKD EPI seems to be less biased in GFR estimation [9]. Analogously, among chronic HF (CHF) patients, especially in those patients with normal or near normal renal function, GFR-EPI allows a more accurate classification of renal function and a better risk stratification [10,11]. However, the reliability of these formulas in the elderly is limited. In patients over 70 years old, the BIS1 equation has been proposed, which is not interchangeable with CKD-EPI or with MDRD. The BIS1 equation gives lower values than CKD-EPI, and classifies patients into a higher level of CKD, mainly when the eGFR is above 30 mL/min/1.73 m^2^ [12].

Creatinine serum levels are used not only to estimate GFR but also to define the occurrence of worsening of renal function (WRF). An increase of creatinine value > 0.3 mg/dL and a decline in the stage of CKD associated with a 25% or greater drop in eGFR from baseline are the criteria indicated by the KDIGO Guidelines to define the presence of a CKD progression [5].

Although creatinine serum levels are the corner stone in order to diagnose the presence of CKD and its progression, their several limitations should be considered. Creatinine serum levels could be influenced by age, diet, gender, body mass and race [6]. Moreover, muscle wasting and cardiac cachexia could lead to a decrease in creatinine levels and thus to an overestimation of GFR as in advanced HF [13]. Moreover, the reduction of GFR occurs when a critical mass of nephrons is lost, due to the increased filtration capacity of the residual nephrons. Consequently, a normal GFR could not reflect an impairment of renal reserve [13]. Finally, GFR is not able to detect early the presence of the pathophysiological conditions leading to renal dysfunction. These limitations are even more relevant in patients with cardiorenal syndrome, in whom an accurate estimation of renal function is essential. Aside to GFR, renal biomarkers should respond to two main clinical needs. From one hand, they should allow to better estimate renal function status and its worsening; from another hand they should more accurately detect those pathophysiological conditions predisposing to acute or chronic worsening of renal function, which can also represent a therapeutic target [4,14].

Cystatin C. Cystatin C, a cysteine proteinase inhibitor, could be a useful tool to overcome some of the limitations related to the estimation of GFR on the basis of creatinine serum levels [15]. It is secreted by all nucleated cells. In comparison with creatinine, it is filtered freely through the glomerulus and then reabsorbed but not secreted by tubular cells. Moreover, Cystatin C serum levels are less dependent on age, body mass, nutritional status and cachexia [15,16]. This is further supported by the more accurate estimation of renal function by Cystatin C when compared with serum creatinine in predicting early postoperative outcomes in advanced HF, i.e., among left ventricular assist device recipients [17]. On the other hand, Cystatin C levels could be increased by some conditions such as inflammation, thyroid dysfunction, obesity and the concomitant use of steroid therapy [18,19,20,21]. In the elderly as well as in patients affected by CVD (coronary artery disease, acute and chronic HF), Cystatin C has shown to be accurate in stratifying the risk of events [22,23,24,25,26]. Actually, the relative high cost in Cystatin C assessment, when compared to creatinine, limits its use in routine clinical practice. According to KDIGO guidelines, the use of Cystatin C should be related to the need of confirming the presence of a renal dysfunction when creatinine alone is biased [5].

Renal functional reserve. Among the possible diagnostic strategies aimed to better evaluate renal function status, the evaluation of renal functional reserve (RFR) could be clinically useful. RFR represents the ability of the kidney to increase GFR and glomerular filtration in response to stimuli in physiological or pathological conditions [27,28]. RFR allows one to increase the glomerular filtration rate of residual nephrons, vicariously, through the lost function and maintenance of the GFR. Because the GFR can remain within normal values until 50% of the nephrons fail are lost, RFR testing can be a sensitive way to more accurately assess functional renal decline, as well as the kidney’s ability to recover after acute injury. However, current methods in order to assess RFR are not easily feasible in clinical practice based on a protein loading followed by the reassessment of creatinine serum levels [27,28]. In the case of reduced RFR, the kidney may be more susceptible to experience renal function worsening.

Microalbuminuria. The assessment of microalbuminuria offers a parameter which reflects the presence of an anomalous renal microcirculation [6] Urine albumin levels are usually very low. This is due to its small size, negative charge and limited tubular absorption. The presence of endothelial dysfunction, inflammation, elevated glomerular pressure and atherosclerosis can cause damage to the glomerular membrane resulting in increased albumin excretion [29,30]. Moreover, in CHF the presence of microalbuminuria could reflect the abnormalities of renal hemodynamics [31]. Consequently, Albuminuria represents a marker of the pathophysiologic background underlying the progression of CKD [29]. In the assessment of renal dysfunction, albuminuria integrates information coming from estimated GFR in order to better stage CKD and stratifying prognosis [5]. Currently, the presence and severity of albuminuria is based on the evaluation of the ratio between urinary albumin and creatinine (UACR). Microalbuminuria is defined as UACR between 30 and 300 mg/g, macroalbuminuria as an UACR > 300 mg/g [30]. Microalbuminuria is highly prevalent in CHF [32], and its presence implies a worse prognosis independently from creatinine serum levels and estimated GFR [33,34].

Tubular biomarkers. Different studies have tested the possible role of tubular biomarkers in predicting the progression of renal dysfunction, particularly in patients affected by CHF. N-acetyl beta glucosaminidase (NAG) is a lysosomal protein of the proximal tubule, excreted into urine in case of tubular damage [35,36]. Kidney injury molecule (KIM1) is a transmembrane glycoprotein which is expressed in proximal tubule cells after hypoxic tubular injury [36]. In patients with CHF, NAG and KIM1 serum levels predict an increased risk of death or HF related hospitalizations, independently from GFR [37,38]. In addition to these biomarkers, alpha-1 microglobulin (A1M), beta-2 microglobulin (B2M), and uromodulin also deserve mention [39]. A1M is a plasma protein synthesized by the liver with an antioxidant effect. Under normal conditions, it is normally filtered by the renal glomerulus and is completely reabsorbed by the renal tubule, but in presence of renal tubular damage it can be detected in the urine [40]. B2M is a nonglycosylated low molecular weight protein present on the surface of all nucleated cells. Normally, only a small fraction of B2M is present in the urine, but when the renal tubules are damaged B2M concentrations increase [41]. Uromodulin, also known as Tamm–Horsfall protein, is a protein produced only by the kidney and is most present in normal human urine. Its function is not well known, but it seems to be to regulate the transport of ions and protect against the formation of kidney stones, infections and kidney damage [42]. High levels of A1M and low levels of uromodulin in the urine are associated with a greater risk of cardiovascular disease and mortality, independently from GFR and albuminuria, whereas no significant association was found between B2M and prognosis [39]. However, the causal link is not well known.

Galectin-3. Aside from microalbuminuria, Galectin-3 (Gal-3) could represent a biomarker able to give information about the pathophysiological background underlying renal dysfunction and its progression in patients with CVD [43,44,45]. Gal-3 is a beta galactoside binding lectin, expressed in various tissues and cell types and detectable inside and outside cells [46,47]. Its main pathophysiological role has been related to its capacity of promoting fibrosis [46,47]. After being released by the activated macrophages, Gal-3 induces fibroblast proliferation as well as the activation and deposition of collagen in the extracellular matrix [46,47]. At the cardiac level, this action can promote cardiac remodeling and progression of heart failure [48,49,50,51]. Beside cardiac effects, experimental data demonstrated that Gal-3 is also involved in renal fibrosis and dysfuncion [52]. In humans, Gal-3 levels are correlated with GFR, but higher Gal-3 plasma galectin-3 levels are also associated with a greater risk of developing incident CKD [53,54]. Analogously, in CHF patients, higher Gal-3 serum levels are not only strongly associated with lower GFR [55] and microalbuminuria [43], but also with an increased risk renal function worsening [44]. Patients with high Gal-3 showed a steeper decline of GFR during a three year follow-up and this was even more evident in patients with apparently preserved renal function, thus strengthening the possible role of this biomarker in early detection of renal dysfunction. The presence of high Gal-3 could reflect the pathophysiological background leading to the reduction of nephrons, and, as a consequence, its greater serum levels could precede the decline of GFR. In the future, Gal-3 could play a role not only as a marker of risk for cardiorenal syndrome progression, but also as a therapeutic target, as suggested by some preliminary data [56].

## 3. Renal Biomarkers in Cardiovascular Patients with Acute Kidney Injury

In acute settings, the term acute kidney injury (AKI) has recently replaced the term acute renal failure. It includes both injury (structural damage) and impairment (loss of function) and it is defined as an abrupt (within hours) decrease in kidney function [57,58]. Among patients hospitalized for CVD, around 25% experience AKI (from 15–30% among patients with acute coronary syndrome (ACS) up to 47% among patients with acute decompensated heart failure) [59,60,61,62,63]. Twenty percent of patients with AKI, 1% to 3% of those with HF or ACS and around 13% of patients with cardiogenic shock need dialysis (AKI-D) [63].

The greater is the severity of underlying CKD the higher is the risk of AKI, as a consequence of a reduced renal reserve and impaired ability of the kidneys to respond to stress [64,65]. Aside from underlying CKD, the other risk factors for AKI are older age, hypertension, diabetes mellitus, sepsis, as well as the use of iodinated radiocontrast material during cardiovascular interventional procedures [64,65,66,67,68]. Furthermore, particularly in patients with HF, renal hemodynamic factors play also a key role in determining AKI [69,70]. GFR is maintained in a normal range by autoregulation mechanisms able to overcome also a significant reduction in cardiac output. In presence of renal hypoperfusion, renin–angiotensin–aldosterone (RAAS) [69] and sympathetic nervous system [71] activation keep an adequate GFR by modulating arteriolar tone both at glomerular and tubular. Only when these mechanisms are exhausted [72] or in presence of a RAAS inhibition [69] does GFR decline with cardiac output. The other hemodynamic condition which can lead to the acute worsening of renal function is the high central venous pressure [70,73,74], which is responsible for an impaired GFR through an increase in interstitial pressure and a reduction of artero-venous gradient [74]. Finally, it is worth noting that AKI could also be the consequence of tubular hypoxia and acute tubular necrosis [75]. Tubular cell damage is most likely to precede glomerular damage.

Actually, the diagnosis of AKI is based on the changes of creatinine serum levels and on the rate of urinary volume [10]. However, the rise of serum creatinine generally follows the tubular damage [4]. In fact, the rise of serum creatinine is generally delayed, and the changes of GFR do not allow an accurate estimation of the severity and timing of AKI. For this reason, over the last years, new biomarkers have been studied in order to earlier detect tubular damage and renal injury [4].

Neutrophil gelatinase associated lipocalin (NGAL), NAG and KIM-1. NGAL is a small protein freely filtered through the glomerulus and completely reabsorbed in the proximal part of the tubule. It is produced by the kidney and other organs and, in normal conditions, its urinary and blood concentrations are very low [76]. When NGAL cannot be completely reabsorbed, due to tubular damage, its urinary levels increase and precede the rise of serum creatinine by at least 24 h, as the other tubular markers [77]. In some studies, enrolling the acute decompensated HF patients, NGAL has been found to be associated with the occurrence of worsening of renal function and with adverse clinical outcome [78,79]. Moreover, NAG and KIM-1 have been tested also in the acute setting among patients affected by CVD [36,80]. In AKI patients, NAG levels rise and are associated with worse prognosis [4]. In the intensive care setting, NAG is related to the risk of acute kidney injury [81]. KIM1 urinary levels increase almost one day before the increase in serum creatinine, with a high sensitivity in the early detection of AKI [38]. A study by Solkoski et al. [82] has shown that in patients with AHF, an increase in biomarkers of tubular damage, especially urinary NGAL and KIM1, is predictive of WRF development and can identify those subjects with greater risk of post-discharge mortality early. However, the clinical usefulness of these markers in clinical practice is still debated, due to the most recent findings which have shown that NGAL levels do not present a greater accuracy than creatinine levels in detecting patients with WRF and/or worse prognosis [82,83,84,85,86]. Finally, the use of NGAL presents some limitations related to a non-univocal sampling frequency and to the influence on its levels by confounding conditions such as sepsis, inflammation, anemia, hypertension, hypoxemia and cancer [76].

Fatty acid-binding proteins (FABPs). FABPs are proteins that bind free fatty acids [87]. In the kidney, liver specific FABP (FABP-1) and heart specific FABP (FABP-3) have been respectively expressed in the proximal and in the distal tubule [88]. Urinary FABP-1 and FABP-3 levels have been associated with ischemic tubular injury and risk for AKI [89]. Moreover, in chronic HF patients, high values of FABP-3 are associated with a greater risk of cardiovascular events [88,89]

G1 cell cycle arrest biomarkers. Several complex cellular and molecular pathways, such as those involving endothelial, epithelial, inflammatory, and interstitial cells, influence the occurrence of AKI. These mechanisms include cell cycle, immunity, inflammation, and apoptosis pathways. Recently two urinary markers of cellular stress in the early phase of tubular cell injury have been proposed, i.e., tissue inhibitor of metalloproteinase 2 (TIMP-2) and insulin-like growth factor-binding protein 7 (IGFBP7) are markers caused by a variety of insults (inflammation, ischemia, oxidative stress, drugs, toxins and ultraviolet radiation) [90,91]. Therefore, both markers are involved in the process of G1 cell-cycle arrest during the very early phases of cell injury that prevents cells from dividing in the case of damage to the DNA until such damage can be repaired before resulting in the cell’ s demise or senescence [92]. Importantly, both biomarkers could represent alarm proteins for tubular damage [92,93].

The usefulness of the measurements in series of biomarkers of tubular damage has been retrospectively evaluated in the patients enrolled in the Sapphire Group study [94]. The results highlighted how TIMP2 and IGFBP7 are useful in predicting AKI in stadium 2–3 in the first 7 days of hospitalization in the Unit of Intensive Care. Their product at baseline, after 12 and 24 h and up at 3 days, is independently associated with the occurrence of AKI. In particular, three consecutive values below 0.3 ng/mL are associated with a very low incidence of stage 2–3 AKI in the next 7 days, whereas values above 2 ng/mL are associated with an increased risk of AKI (up to 94.4%) [95]. Moreover, the accuracy of TIMP2 and IGFBP7 in predicting the occurrence of AKI is greater than those of KIM-1, NGAL, L-FABP, IL-18, or Cystatin C [96,97,98]. Finally, results of the PrevAKI randomized trial have shown that the product of IGFBP7 and TIMP-2 by guiding KDIGO recommended monitoring and treatments can allow a reduction in the incidence of post-cardiac AKI [99].

## 4. Cardiac Biomarkers in Kidney Diseases

The aim of this review was mainly to focus on the renal biomarkers reflecting the severity of CRS. However, some aspects concerning cardiac biomarkers should be considered. Brain natriuretic peptide (BNP) and amino-terminal pro-BNP (NT-proBNP) are biomarkers which are useful for diagnosis and prognostic stratification of HF patients [100]. It is worth noting that their serum levels, particularly for NT-proBNP, could be influenced by renal function, i.e., the more severe the renal dysfunction, the higher the serum levels [100]. The mechanisms of this relationship are not fully elucidated, but natriuretic peptides continue to be associated with a worse prognosis also in patients with CKD [100]. Moreover, an approach based on the evaluation of BNP and bio impedance analysis has been proposed in order to better tailor diuretic therapy and reduce the occurrence of AKI [101]. In chronic HF, NT-proBNP is associated with WRF, but this association is not independent from the other clinical and echocardiographic parameters and Galectin -3 [44].

Although high troponin levels are associated with a worse prognosis also in type 3 and 4 CRS, it should be considered that most patients with more severe CKD present troponin levels above the normal range [100]. Moreover, in acute HF troponin as well as NTproBNP fail to be independently associated with WRF [102].

## 5. Biomarkers of Renocardiac Syndrome

Among the cardiorenal biomarkers, those related to the end-stage renal disease (ESRD) should be also high lightened [103]. ESRD could determine the occurrence of cardiac structural and functional abnormalities until the onset of heart failure. Hemodynamic factors related to fluid overload as well as to a high flow state, related to arterio-venous fistulas, can promote the eccentric remodeling of the left ventricle [104]. On the other hand, an increased afterload, due to higher arterial systemic resistances and/or a reduced arterial compliance, can promote a ventricular concentric remodeling. Finally, myocardial fibrosis could be enhanced by nonhemodynamic factors such as uremic toxins, oxidative stress, inflammatory status, hyperparathyroidism, hypovitaminosis D and hyperphosphatemia [103,105].

The rise of the uremic toxins is the consequence of the progressive loss of the ability to eliminate both the substances coming from the human metabolism and those of its symbiont, the intestinal microbiota [103]. The intestinal microbiota, in patients suffering from chronic kidney disease, is completely different from that of the healthy subject: this imbalance is called “dysbiosis” [106]. With the decline of renal function, the colon assumes the role of excretion organ. Urea excretion leads to colon pH increase, thus favoring the growth of urease-positive species, which are responsible for the conversion of urea into ammonia. The consequent degradation of the protective mucus layer and alteration of intestinal permeability causes the passage of bacterial material through the mucosa and the activation of a chronic local and systemic inflammatory mechanism. Moreover, bacteria use amino acids not with an anabolic function, but for energy purposes, resulting in the production of uremic toxins.

Among the uremic toxin of intestinal derivation, p-cresyl sulfate (PCS), indoxyl sulfate (IS) and trimethylamine N-oxide (TMAO) are those with larger evidence [107,108,109,110,111,112]. PCS and IS derive from the degradation of aromatic amino acids, such as tryptophan, phenylalanine and tyrosine, whereas TMAO come from the catabolism of products essentially of animal origin, containing choline, phosphatidylcholine, carnitine and betaine. In CKD patients, PCS and IS reach levels even 100 times higher than in healthy subjects. These substances are characterized by a pro-fibrotic, pro-inflammatory and oxidative stress induction both at the renal and cardiovascular level [106,110,111]. Moreover, they are able to promote cardiac hypertrophy, thus further favoring the progression of cardiac dysfunction in ESRD [113].

The cardiovascular relevance of IS and PCS is supported by their association with a worse prognosis in patients with CVD and renal impairment [112]. IS and PCS are difficult to be removed by the conventional dialysis due to their substantial protein-binding capacity [110]. Targeting these toxic solutes may represent therapeutic opportunity in order to attenuate CRS progression [109,112].

## 6. Conclusions

In patients affected by CVD and HF, CKD is highly prevalent. Its presence as well as its worsening is associated with a worse prognosis. Therefore, an accurate renal function evaluation plays a key role in order to stratify patients’ prognosis. GFR estimation based on serum creatinine levels is the easiest way to assess overall kidney function in clinical practice and it is routinely used. However, several limitations in the use of serum creatinine exist in chronic as well as in acute settings. To overcome some of the caveats related to creatinine, new markers have been studied in order to better asses the severity of renal dysfunction, to detect patients at higher risk of renal function worsening as well as to more accurately identify patients prone to developing AKI. Finally, new biomarkers have been proposed in order to evaluate patients with end-stage renal disease prone to experiencing a worsening of cardiac function. Future studies should help to understand if these biomarkers could be not only prognostic markers but also the basis or target for new therapeutic approaches.

## Figures and Tables

**Table 1:** Characteristics of the main renal biomarkers indicative of cardiorenal and renocardiac syndrome progression.

Marker	Biological Function	Dosage Site	Clinical Setting	Proposed Cut-Offs
Cystatine C	Protease Inhibitor	Blood	AKI	>1.5 mg/L (blood)
Galectin 3	Lectin function: cell growth and differentiation	Blood	CHF	>13.5 for GFR >60 >18.1 for GFR < 60
NGAL	Proliferative and antiapoptotic action	Urine and Blood	AKI CHF	>50 microg/L (urine)
NAG	Lysosomial Enzyme	Urine	AKI CHF	>50 mU/mg (urine)
KIM 1	Tubular Regeneration	Urine	AKI CHF	From 10 to 15 mg/ng (urine)
FABPs	Free Fatty acids binding proteins	Urine	AKI	>15 microg/g Cr (urine)
TIMP2 & IGFBP7	G1 Cell cycle arrest markers	Urine	AKI	>0.3
P-Cresyl Sulfate (PCS) Indoxyl Sulfate (IS)	Uremic Toxins	Blood	Reno-cardiac syndrome	100 times higher than in healthy subjects

NGAL: N-acetyl beta glucosaminidase. NAG: N-acetyl beta glucosaminidase. KIM 1: Kidney injury molecule. FABPs: Fatty acid-binding proteins; TIMP2: Tissue inhibitor of metallo-proteinase 2; IGFBP7: insulin-like growth factor–binding protein 7; AKI, acute kidney injury; CHF, chronic heart failure; GFR, glomerular filtration rate.
